# Tobacco Control Continues to Work as Smoking Prevalence Declines: Combating Manufactured Uncertainty: Author Response

**DOI:** 10.1093/ntr/ntac142

**Published:** 2022-06-09

**Authors:** Emily Banks, Miranda Harris, Amelia Yazidjoglou, Melonie Martin, Eryn Newman, Laura Ford, Robyn M Lucas

**Affiliations:** National Centre for Epidemiology and Population Health, Australian National University, Canberra, Australian Capital Territory, 2601, Australia; National Centre for Epidemiology and Population Health, Australian National University, Canberra, Australian Capital Territory, 2601, Australia; National Centre for Epidemiology and Population Health, Australian National University, Canberra, Australian Capital Territory, 2601, Australia; National Centre for Epidemiology and Population Health, Australian National University, Canberra, Australian Capital Territory, 2601, Australia; Research School of Psychology, College of Health and Medicine, Australian National University, Canberra, Australian Capital Territory, 2601, Australia; National Centre for Epidemiology and Population Health, Australian National University, Canberra, Australian Capital Territory, 2601, Australia; National Centre for Epidemiology and Population Health, Australian National University, Canberra, Australian Capital Territory, 2601, Australia

Our review of the current evidence demonstrates that, in countries with high-quality tobacco control, falling smoking prevalence is generally accompanied by — and a natural consequence of — a general smoker population that is less dependent on smoking and more motivated and better able to quit.^1^ Hence, tobacco control reduces smoking prevalence and fosters a smoking population more amenable to evidence-based interventions.^1^

Belief underpins action. The generation of hypotheses and their falsification or verification based on evidence is crucial to ensuring scientific progress, and to avoiding the harm that results from holding, and acting on false beliefs. Seeking to perpetuate uncertainty about a hypothesis that has been falsified is neither scientifically prudent nor benign. Charles Darwin said: “I have steadily endeavored to keep my mind free so as to give up any hypothesis, however much beloved….as soon as facts are shown to be opposed to it.”^2^ The evidence is clearly opposed to the hardening hypothesis and the belief that population hardening occurs with falling smoking prevalence. As our paper outlines, active steps are required to shift the discourse to accepting the reality of the “softening” of the general smoking population as smoking prevalence declines.^1^

Influencing science aims to change the knowledge upon which decision makers set policy and upon which action rests. The creation of doubt regarding research findings that are not supportive of tobacco industry goals is part of the World Health Organization (WHO) taxonomy of tactics of the tobacco industry to further its interests.^3,4^ A common way of doing this is for individuals connected with the industry to write a letter to the editor critical of a peer-reviewed publication they are seeking to discredit.^5^ Regardless of the response of the authors, this letter is then cited by industry, and others supporting its interests, as evidence that the original paper is flawed or discredited. Letters to the editor are not subject to peer review and can, therefore, provide a less scrutinized means of allowing industry and those it funds to publish in scientific journals.^5^

The tobacco industry has a vested interest in perpetuating the myth that tobacco control becomes less effective as smoking prevalence falls. This myth can be used to undermine and dampen policy support for ongoing tobacco control, including in settings where it has been successful to date, and is also used as grounds for the promotion of novel tobacco products. As noted in our paper, this myth also has the effect of focusing attention away from effective population-level strategies and onto addiction to cigarettes as an immutable attribute of a person, rather than an intentional, industry-generated phenomenon.^1^ The myth also runs counter to the fact that smokers continue to quit and most often do so unaided. “Exposing myths” is a WHO-recommended strategy to combat this type of tobacco industry activity, as part of the implementation of the Framework Convention on Tobacco Control.^4^

Our review was conducted according to current best practices and underwent extensive independent peer and editorial review. The authors are independent of the tobacco and related industries. In order to avoid tobacco industry interference, in keeping with Article 5.3 of the WHO Framework Convention on Tobacco Control, our research group does not engage with the tobacco industry and those funded by it. We are therefore precluded from responding specifically to the points raised in the letter of consultants Steffensen et al., since they report having received funding from RAI Services Company, a wholly owned subsidiary of British American Tobacco.^6^

There is a fundamental and irreconcilable conflict between the interests of public health and those of the tobacco industry.^7^ The tobacco industry has a long history of large-scale systematic and conscious undermining of the scientific process and of research findings that do not support its agenda, and there is clear evidence of bias in research and commentary published by those funded by industry.^3,5^ The scientific community, including scientific journals, must protect itself and the integrity of science from these efforts and it is of paramount importance that journals avoid providing a platform for tobacco industry activities, including efforts to discredit high-quality peer-reviewed research. The fact that *Nicotine and Tobacco Research* does not publish material from tobacco industry employees is an important part of this. However, the use of third parties, including consultants, is one of the many WHO-recognized tobacco industry tactics used to further their interests.^3^*Nicotine and Tobacco Research*, the research it publishes, and the community it serves remain vulnerable to this approach. We join the calls made by others for *The Society for Research on Nicotine and Tobacco* and *Nicotine and Tobacco Research* to include individuals receiving funding from the tobacco industry, as well as tobacco company employees,^8^ among those who are not allowed to publish in the journal, in keeping with the policies of many other journals.^9^

## Supplementary Material

ntac142_suppl_Supplementary_Taxonomy-form
